# HNRNPA2B1 stabilizes NFATC3 levels to potentiate its combined actions with FOSL1 to mediate vasculogenic mimicry in GBM cells

**DOI:** 10.1007/s10565-024-09890-5

**Published:** 2024-06-11

**Authors:** Hanting Wang, Yiwen Shi, Xinxin Zhou, Lu Zhang, Aodan Yang, Dabo Zhou, Teng Ma

**Affiliations:** 1 Department of Neurobiology, School of Life Sciences, China Medical University, Shenyang, 110122 China; 2 Liaoning University of Traditional Chinese Medicine, Shenyang, 110034 China; 3 The First Clinical College of China Medical University, Shenyang, 110002 China; 4 School and Hospital of Stomatology, China Medical University, Shenyang, 110002 China

**Keywords:** Glioblastoma (GBM), Vasculogenic mimicry (VM), Transcription factor, Xenograft, Gene expression

## Abstract

**Background:**

Vasculogenic mimicry (VM) is an enigmatic physiological feature that influences blood supply within glioblastoma (GBM) tumors for their sustained growth. Previous studies identify NFATC3, FOSL1 and HNRNPA2B1 as significant mediators of VEGFR2, a key player in vasculogenesis, and their molecular relationships may be crucial for VM in GBM.

**Aims:**

The aim of this study was to understand how NFATC3, FOSL1 and HNRNPA2B1 collectively influence VM in GBM.

**Methods:**

We have investigated the underlying gene regulatory mechanisms for VM in GBM cell lines U251 and U373 *in vitro* and *in vivo*. In vitro cell-based assays were performed to explore the role of NFATC3, FOSL1 and HNRNPA2B1 in GBM cell proliferation, VM and migration, in the context of RNA interference (RNAi)-mediated knockdown alongside corresponding controls. Western blotting and qRT-PCR assays were used to examine VEGFR2 expression levels. CO-IP was employed to detect protein–protein interactions, ChIP was used to detect DNA–protein complexes, and RIP was used to detect RNA–protein complexes. Histochemical staining was used to detect VM tube formation in vivo.

**Results:**

Focusing on NFATC3, FOSL1 and HNRNPA2B1, we found each was significantly upregulated in GBM and positively correlated with VM-like cellular behaviors in U251 and U373 cell lines. Knockdown of NFATC3, FOSL1 or HNRNPA2B1 each resulted in decreased levels of VEGFR2, a key growth factor gene that drives VM, as well as the inhibition of proliferation, cell migration and extracorporeal VM activity. Chromatin immunoprecipitation (ChIP) studies and luciferase reporter gene assays revealed that NFATC3 binds to the promoter region of VEGFR2 to enhance VEGFR2 gene expression. Notably, FOSL1 interacts with NFATC3 as a co-factor to potentiate the DNA-binding capacity of NFATC3, resulting in enhanced VM-like cellular behaviors. Also, level of NFATC3 protein in cells was enhanced through HNRNPA2B1 binding of NFATC3 mRNA. Furthermore, RNAi-mediated silencing of NFATC3, FOSL1 and HNRNPA2B1 in GBM cells reduced their capacity for tumor formation and VM-like behaviors *in vivo*.

**Conclusion:**

Taken together, our findings identify NFATC3 as an important mediator of GBM tumor growth through its molecular and epistatic interactions with HNRNPA2B1 and FOSL1 to influence VEGFR2 expression and VM-like cellular behaviors.

**Graphical Abstract:**

1. NFATC3 binds to the promoter region of VEGFR2 to enhance VEGFR2 gene expression which leads to an increase in VM of GBM.

2. FOSL1 interacts with NFATC3 to further facilitate VEGFR2 gene expression and VM.

3. HNRNPA2B1 enhances NFATC3 mRNA stability to increase VEGFR2 expression and VM.

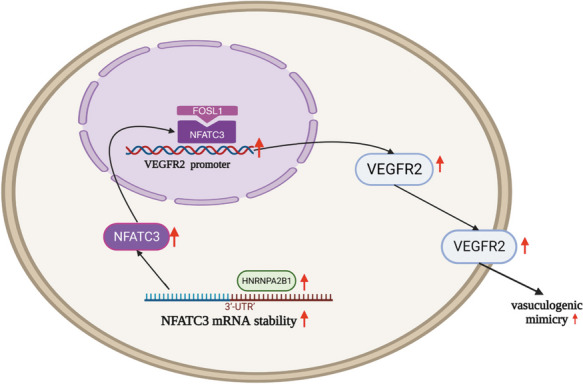

**Supplementary Information:**

The online version contains supplementary material available at 10.1007/s10565-024-09890-5.

## Introduction

Gliomas are one of the most common primary malignant brain tumors diagnosed in adults and arises as a consequence of uncontrolled growth of glial cells or their precursors. The World Health Organization (WHO) classifies gliomas into four grades based upon growth patterns and detected mutations to isocitrate dehydrogenase, among which glioblastoma (GBM) is recognized as the highest grade (IV) glioma (Lapointe et al. 2018; Louis et al. 2016). GBM is an invasive solid tumor, and the main treatment methods include surgery, radiotherapy, temozolomide chemotherapy (Lapointe, et al. 2018; Weller et al. 2017). In addition, GBM treatments with the anti-vascular drug bevacizumab also show promise, and patients prescribe with this treatment show reductions in peritumor edema and corticosteroid use, and a significant increase in progression-free survival, yet overall survival did not improve. The median survival time of GBM patients is less than two years, while the five-year survival rate is only 5% (Lofgren et al. 2022; Yang et al. 2022).

In GBM, tumors can form unique patterns of tube-like formations in which tumor cells replace the endothelium and form a structure that appears similar to blood vessels, a cellular phenomenon described as vasculogenic mimicry (VM). The cellular features of VM include the prominence of matrix protein-rich cells that do not express endothelial markers such as CD31 and CD34, while showing positive histochemical staining for periodic acid–Schiff (PAS) that detects the abundant carbohydrate stores (predominantly glycogen) in tumor cells, and the presence of blood components (Maniotis et al. 1999; Xiang et al. 2018, Buccarelli et al. 2022). VM also leads to the creation of a microenvironment that can deliver oxygen and nutrients to tumors, which is associated with the development and metastasis of a variety of tumors, including gliomas, and is ineffective against anti-angiogenic therapy (Huang, et al. 2023). Indeed, patients with tumors that feature VM have worse clinical outcomes that those that do not feature VM (Luo et al. 2020). The molecular and cellular mechanisms of VM remain poorly characterized.

One molecular player associated with tumor cells is nuclear factor of activated T cells (NFAT), a transcriptional activator that binds promoter regions within the interleukin-2 (IL-2) gene (Serfling et al. 1989). Members of the NFAT family of proteins (including NFATC1-5 proteins) share a moderately conserved NFAT homology domain (NHD), a highly conserved Rel-homology domain (RHD) and calcineurin-docking site (CDS) regions, with the exception of NFAT5 that does not contain a CDS polypeptide region (Qin et al. 2014). Also, NFAT binds DNA through the RHD region (Jain et al. 1995) and functions as a transcription factor to promote the growth and metastasis of a variety of tumor cells, including breast cancer (Goshima et al. 2019), pancreatic carcinoma (Tong et al. 2022) and GBM (Chigurupati et al. 2010). Interestingly, one study showed that only NFATC3 was highly expressed in a variety of glioma cells (Ellert-Miklaszewska et al. 2021). Evidence in the GEPIA database shows NFATC3 is prominently expressed in GBM. Furthermore, our previous study demonstrated that NFATC3 functions as a transcription factor to affect endothelial cells function and regulate blood–brain barrier permeability (Ning et al. 2022). Therefore, we reasoned that NFATC3 could be an important molecular player in GBM. Consistent with this hypothesis, mutations in NFATC3 are detected in epithelioid glioblastoma, as reported in a recent study (Lin et al. 2022). These lines of evidence could suggest that NFATC3 is relevant to VM in GBM.

The transcriptional activity of NFATC3 involves interactions with combinations of chaperone factors to orchestrate gene expression. For example, the RHD of NFATC3 has a specific binding site for FOS and JUN (Qin et al. 2014). Furthermore, Activator protein 1 (AP1), a complex comprising FOS and JUN, acts a chaperone for NFAT that mediate its roles in T cell activation. There, AP1 dimers and NFAT interact with the DNA binding site of NFAT and form a quaternary complex that regulates the expression of multiple downstream protein-coding genes (Macian 2005; Bengsch and Wherry 2015). Focusing on the AP1 component FOSL1 (Duncan 2022), we find that its expression was the highest among multiple AP1 members detectable in GBM. Furthermore, data from the STRING database outlines a putative interrelationship between FOSL1 and NFATC3. Notably, data from the GEPIA database shows that mortality rates for patients with GBM were positively correlated with high levels of FOSL1. The functional interaction between NFATC3 and FOSL1, and their potential to modulate VM in GBM has so far not been reported.

High levels of NFAT protein in GBM (Qin et al. 2014) may be attributable to the relative abundance of this mRNA species within tumor cells. In the context of mRNA regulation, members of the HNRNP family of proteins are prominent in the nucleus and cytoplasm, and participate in the maturation and transport of hnRNA, and regulate its translation and other processes. These HNRNPs recognize and bind different sequences to elicit their gene regulatory functions (Liu and Shi 2021). For example, HNRNPA1 recognizes the RNA sequence GUAGUAGU to initiate alternative splicing (Huelga et al. 2012), while HNRNPA2B1 activity is associated with the presence of UAGGG sequences (Ray et al. 2013) and GA-rich regions of mRNAs to regulate their stability (Kasim et al. 2014). In studies of GBM cells, it was found that HNRNPA2B1 can be used as a potential marker to assess the severity of GBM, as well as its proliferative potential and the propensity of GBM cells for drug resistance (Deng et al. 2016; Yin et al. 2021). Indeed, data from the UCSC database on NFATC3 shows that multiple UAGGG sequences can be found within the its 3’UTR region, which suggests these could serve as binding sites for NFATC3, FOSL1 and VEGFR2, as predicted by starbase3.0 database (Li et al. 2014). These lines of evidence suggest that HNRNPA2B1 could be relevant to NFATC3 and its downstream impacts in GBM and VM.

In addition to NFATC3 and FOSL1, another important player for tumor invasiveness in GBM is VEGFR2, a vascular endothelial growth factor also known as KDR. VEGFR2 is expressed in the vascular endothelium and promotes endothelial proliferation and recruitment and is recognized as a marker of tumor VM. Indeed, VEGFR2 is significantly abnormally high expressed in GBM and promotes VM formation (Yao et al. 2013; Cui et al. 2023; Mao et al. 2015). Guided by these collective insights, we were motivated to investigate the putative functions for NFATC3, FOSL1 and HNRNPA2B1 on VM formation and establish its potential interactions in the context of VEGFR2 expression. Our goal was to clarify the potential interactions between these molecules in GBM so as to better understand both the diagnosis and biological basis for anti-vascular treatment of GBM.

## Materials and methods

### Access to public data resources

The mRNA levels for NFATC3, FOSL1 and HNRNPA2B1 documented in different tissue samples from grades of glioma were obtained from the gene expression collections of the CGGA database (http://www.cgga.org.cn/index.jsp). Data for steady-state protein expression levels and survival rates of glioma patients was obtained from the GEPIA database (http://gepia.cancer-pku.cn/). The cut-off values for NFATC3, FOSL1 and HNRNPA2B1 protein expression levels were set at 50%. All mRNA sequences as well as VEGFR2 promoter sequences were obtained from the UCSC database (https://genome.ucsc.edu/), version GRCh38/hg38. The binding sites for NFATC3 and FOSL1 within the VEGFR2 promoter region were predicted using JASPAR (https://jaspar.genereg.net/). Predictions for protein–protein interaction were derived from data from the string database (https://cn.string-db.org/) and GeneMANIA (https://genemania.org/). Information from protein-to-mRNA binding predictions, as well as correlations of interactions between HNRNPA2B1 and NFATC3/FOSL1/VEGFR2 were sourced from Starbase3.0 (https://rnasysu.com/encori/) as well.

### Cell lines and cell culture

Normal human astrocytes (HA) were sourced from ScienCell (Cat. No.1800, CA, USA) and used as controls for cell culture studies and were maintained in astrocyte medium (ScienCell, CA, USA). The human glioblastoma cell line U251 was sourced from Wuhan Pricella, Cat. No.: CL- 0237. The human glioblastoma cell line U373 cell line was obtained from Genesky Biotechologies (Cat. No. 23B0531C). Cultures of U251 and U373 cells were maintained in DMEM medium (Corning, NY, USA) containing 10% FBS (Gibco, NY, USA). All cells were cultured in an incubator maintained at 37℃ and 5% CO2.

### Quantitative real-time reverse transcription PCR (qRT-PCR)

Trizol reagent (Invitrogen, Inc.) was used to extract RNA from samples of glioma cell lines and glial cells. RNA extraction was performed according to instructions supplied by the manufacturer. Complementary DNA (cDNA) samples were synthesized by reverse transcription reactions using a RT reagent Kit with gDNA Eraser (Takara, Japan), and qRT-PCR was performed with TB Green Premix Ex Taq II (Takara, Japan). Steady-state levels of mRNA from samples were measured using a Roche LightCycler 480. A list of primers used is provided in Supplementary Table S1A.

### Cell transfection

Plasmids encoding short hairpin RNAs (shRNAs) that target NFATC3 and FOSL1 for silencing by RNA interference (RNAi) were constructed on pGPU6/mCherry/Puro vector and pGPU6/GFP/Neo (GenePharma, Shanghai, China) and were referred to as NFATC3(-) and FOSL1(-) in this study. HNRNPA2B1 knockdown siRNA was obtained from Sangon Biotech (Shanghai, China), and referred to here as HNRNPA2B1(-). Non-targeting sequences were used as corresponding NC groups. Lipofectamine 3000 and P3000 (Life Technologies, Carlsbad, CA, USA) transfection reagents were used to introduce plasmids into cells, following which cells were cultured in the presence of puromycin and G418 to select stably transfected cells. Primers, probes, shRNA and siRNA sequences used are presented in Supplementary Table S1B. Transfection efficiencies are shown in Supplementary Fig. S1.

### Western blotting

Protein extraction was conducted using RIPA (Beyotime, Shanghai, China) lysis buffer containing PMSF (100:1; Beyotime, Shanghai, China). Protein concentrations for lysate preparations were quantified using a BCA Protein Assay Kit (Beyotime, Shanghai, China). Forty micrograms of a given protein sample was loaded into a well of a 10% SDS-PAGE gel for electrophoresis, following which size-separated proteins for the sample were transferred onto PVDF membranes (Millipore, Billerica, MA, USA). Blots were pre-incubated in TTBS wash buffer containing 5% skim milk, followed by incubation with primary and secondary antibodies. The antibodies used were: GAPDH (1:5000; Proteintech, Wuhan, China), NFATC3 (1:2000; Proteintech, Wuhan, China), FOSL1 (1:2000; Cloud-Clone, Wuhan, China), HNRNPA2B1 (1:2000; Proteintech, Wuhan, China) and VEGFR2 (1:2000; Proteintech, Wuhan, China).

### Cell proliferation assay

A total of 4 × 10^3^ cells were inoculated in wells of 96-well plates, and five duplicate wells were set in each group. After 24 h of culture, 100 μl of fresh DMEM medium was replaced and 10 μl of CCK-8 (Dojindo, Kumamoto, Japan) was added. Then, the culture was continued for 2 h at 37℃ with 5% CO_2_. One hundred microliters of fresh DMEM medium and CCK-8 were both also added into each of five blank wells to represent a blank control group. The absorbance at 450 nm for each well was measured using a multiplate reader, with signals measured as the absorbance of each well subtracted by the average absorbance of the blank hole in the experiment. Quantification of cell proliferation was defined as the percentage of the actual absorbance relative to the control group.

### Cell migration assay

Prior to placement of Transwell chambers into wells of 24-well plates that contained cells, 500 μl of culture medium supplemented with 10% FBS was added. Next, 200 μl of FBS-free culture medium was mixed with 2 × 10^4^ cells and added to the upper chamber. The cells were then cultured for 24 h to observe transwell migration. For experiments with U251 cells, such cells were cultured for 48 h. The cells were subsequently fixed by application of 4% paraformaldehyde solution, washed and then stained with Giemsa (Leagene, Beijing, China) overnight. The stained GBM cells were photographed under a microscope with a 200 × field of view. Image J was used for cell counts.

### VM formation assay (Matrigel 3D cell culture)

A final volume of 100 μl Matrigel (BD Biosciences, Bedford, MA, USA) was added to each well of a 96-well plate that was sitting on ice. Air bubbles were removed from each well comprising the cell-matrigel suspension and the culture plate was then returned to the incubator for 30 min. Next, a suspension of 4 × 10^4^ cells in 100 μl of culture medium was inoculated into each well containing matrigel and cultured in an incubator at 37 ℃ for 8 h. An inverted microscope was then used to photograph each well at 200 × magnification. Image J was used to count the number of vascular structures.

### RNA half‐life assay

At the commencement of the assay, the media of cultured cells in a given well was replaced with fresh media containing actinomycin D (actD, MP Biomedicals) at a final concentration of 5 mg/ml and incubated for 0, 2, 4, 6, and 8 h. Following incubation times, total RNA was extracted from each group of cells for RT-PCR analysis.

### Chromatin immunoprecipitation (ChIP) and ChIP-qPCR analysis

A ChIP kit (Beyotime, Shanghai, China) was used according to instructions of the manufacturer, as follows. Briefly, cells were crosslinked with 1% formaldehyde. Subsequently, were lysed by IP lysis buffer (Beyotime, Shanghai, China) before sonication. Next, 20 ml of each sonicated sample was used as input control. Antibodies were added to the remaining samples and incubated in tubes on the rotator at 4 °C overnight. Following this, DNA was eluted and un-crosslinked, and the DNA was then purified using the DNA Purification Kit (Beyotime, Shanghai, China), and analyzed using qRT-PCR. The primers used in this study are shown in Supplementary Table S1C.

### RNA immunoprecipitation (RIP)

Cell lysates were collected and 10% of the lysate from each sample was retained as input, as per instructions provided with the RIP kit (BersinBio, Guangdong, China). The remaining lysates were incubated with 3 mg antibodies (HNRNPA2B1 and IgG) overnight at 4 °C with shaking. Next, beads were added to immunoprecipitated RNA–protein complexes the next day and incubated at 4 °C for 1 h. Then, complexes were eluted, the protein was enzymatically digested, and qRT-PCR was performed.

### Dual-luciferase reporter assays

Putative binding sequences within the NFATC3 in the VEGFR2 promoter regions were amplified by PCR and cloned upstream of the pgL3 Luciferase Expression Vector (Promega, Madison, WI, USA) to construct wildtype luciferase reporter vectors. Mutated versions of these luciferase reporter vectors were also generated using a similar approach. NFATC3(-), NFATC3(-), NC and control group GBM cells (U251 and U373) were then transfected with these vectors, respectively. After 48 h, luciferase activity was detected as per the manufacturer’s protocol (Promega, USA), and the signals for firefly luciferase were used as a reference to establish the relative luciferase activity levels of samples for each group. The wildtype and mutant sequences used in this study are displayed in Supplementary Table S1D.

### Co-Immunoprecipitation (CO-IP)

A CO-IP kit (BersinBio, Guangdong, China) was used as per the manufacturer’s protocol, as explained briefly. Cells were gently lysed, then an aliquot of anti-NFATC3 (3 mg per sample) or anti-FOSL1 (4 mg per sample) antibodies was added to their respective reaction tubes and incubated for their prescribed times. Control reactions were incubated with IgG (IgG Group). Next, protein A/G magnetic beads were added to each reaction tube and incubated, following which the beads were washed with chilled wash buffer to remove the non-specifically bound proteins. Proteins were eluted with SDS-PAGE loading buffer and Western blotting was performed to identify the relevant proteins of interest.

### Tumor xenograft implantation in nude mice

Mice for this study were purchased from HFK Bioscience (Beijing, China). Each mouse received a subcutaneous injection of 5 × 10^6^ GBM cells (U251 and U373) on the right flank, and tumors formed after approximately 30 days after cell injection. The tumors were photographed, the lengths of tumors were measured. Tumor volumes were calculated for each sample according to the following formula: volume (mm^3^) = (length × width^2^)/2.

### Histochemical staining

Paraffin-embedded tumor tissues were sectioned at 5 μm thickness. Sections were immunostained for CD31, and periodic acid–Schiff (PAS) staining was also carried out after paraffin-embedded tissue sections were floated onto glass slides, dried, dewaxed and re-hydrated. The sections were counterstained with hematoxylin, subject to dehydration and sealed with a glass cover slip and sealed with neutral gum. Tubular structures that were CD31(-) + PAS( +) by immunostaining and visualized at 200 × magnification were counted.

### Statistical analysis

We repeated each experiment at least three times independently. All data were expressed as the mean ± standard deviation. Statistical analysis was performed using GraphPad Prism 8.0 software. Differences between two or multiple groups were assessed respectively by two-tailed Student’s t test, or one-way ANOVA. The value of *p* < 0.05 was considered statistically significant.

## Results

### NFATC3 was significantly upregulated in GBM cells and promoted VM behaviors

**Fig. 1:**
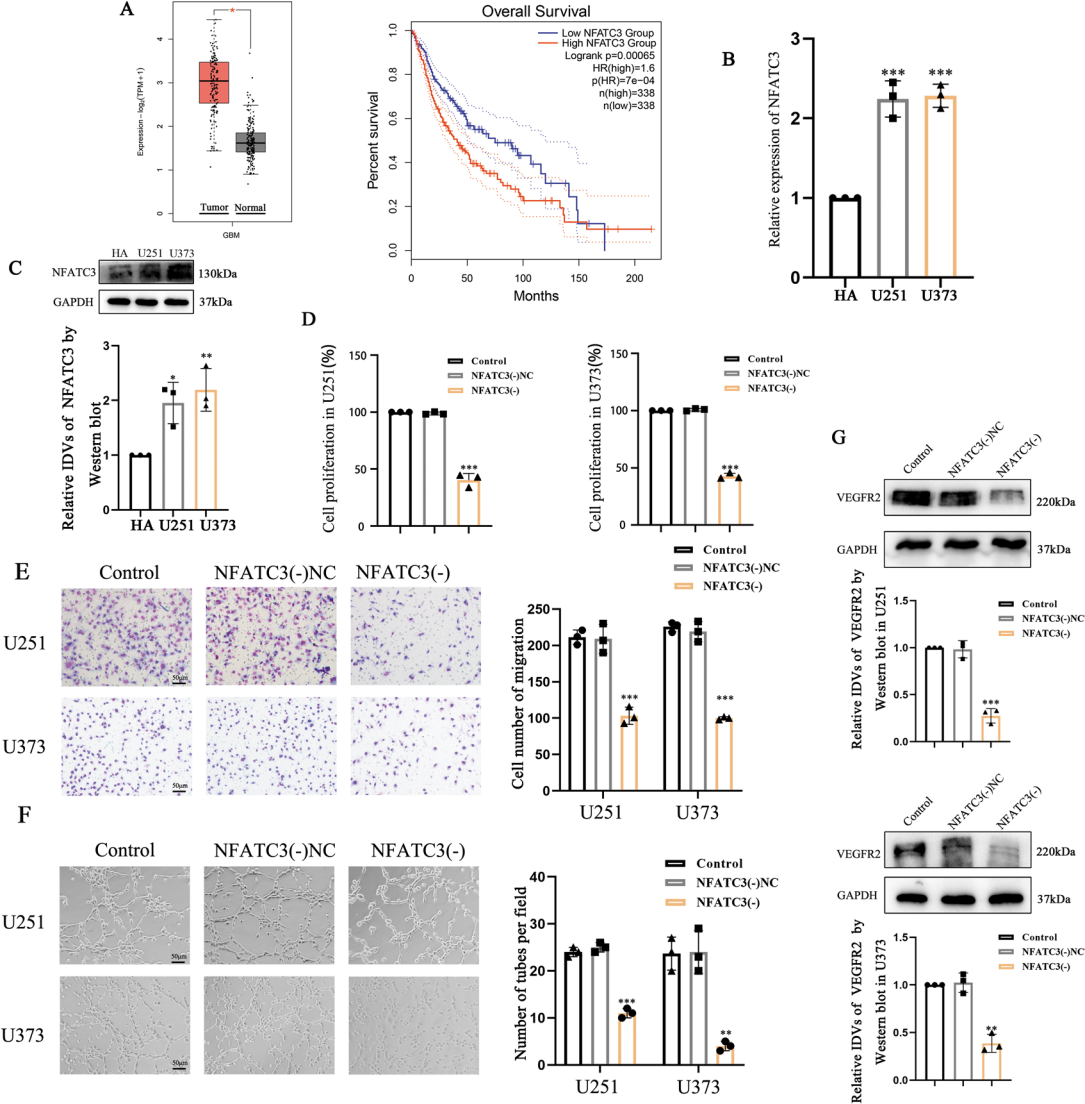
NFATC3 is prominently expressed in GBM cells and promotes VM-like behaviors in GBM cells. **A** Data from the GEPIA database shows elevated NFATC3 expression levels in GBM tissues compared with normal tissues (^*^p < 0.05), while NFATC3 expression levels were negatively correlated with survival time of glioma patients. (*p* < 0.001) **B** Relative expression levels for NFATC3 in HA, U251 and U373 cell lines detected by qRT-PCR (^***^p < 0.001 compared with the HA group). **C** Relative expression of NFATC3 in HA and GBM cell lines detected by Western blotting. GAPDH was used as an internal control. Compared with the HA group, ^*^p < 0.05 and ^**^p < 0.01. **D** Effects of shRNA-mediated NFATC3 knockdown on the proliferative capacity of U251 and U373 cells using a CCK8 assay. Y-axis plots the percentage absorbance relative to control. Compared with the NFATC3(-)NC group, ^***^p < 0.001. **E** Transwell assays to assess cell migration by U251 and U373 following NFATC3 knockdown. The chart represents the number of cells crossed in each well. Compared with the NFATC3(-)NC group, ^***^p < 0.001. **F** Matrigel 3D cell culture assays to observe formation of tube-like structures by GBM cells following NFATC3 knockdown. Compared with the NFATC3(-)NC group, ^**^p < 0.01 and ^***^p < 0.001. **G** Steady state levels of immunoblotted VEGFR2 signal were measured by Western blot. GAPDH was used as an internal control. Compared with the NFATC3(-)NC group, ^**^p < 0.01 and ^***^p < 0.001

We began with a survey of bioinformatic information on NFATC3 expression in various contexts. Data from the CGGA database showed that NFATC3 mRNA levels were positively correlated with the severity of glioma malignancy (Zhao et al. 2021) (Fig. S2A). Higher expression levels for NFATC3 were observed in GBM compared to normal tissue, and high expression of NFATC3 was negatively correlated with overall survival for glioma patients (Fig. 1A). We performed qRT-PCR and Western blotting to confirm that NFATC3 mRNA and protein were highly expressed in GBM cell lines U251 and U373 (Fig. 1B, C). Using a CCK8 assay to measure cell proliferation, we found that shRNA-mediated knockdown of NFATC3 inhibited the proliferation of these two cell lines (Fig. 1D). Transwell assays verified that knockdown of NFATC3 also disrupted their capacity for cell migration (Fig. 1E), while matrigel 3D cell culture assays showed that knockdown of NFATC3 significantly reduced the capacity for both U251 and U373 cell lines to forming formations resembling tubular structures (Fig. 1F). Western blot analysis also showed the knockdown of NFATC3 led to reductions in the steady-state levels of immunoblotted VEGFR2 (Fig. 1G).

### FOSL1 was significantly upregulated in GBM cells and facilitated VM-like behaviors in GBM cells

**Fig. 2:**
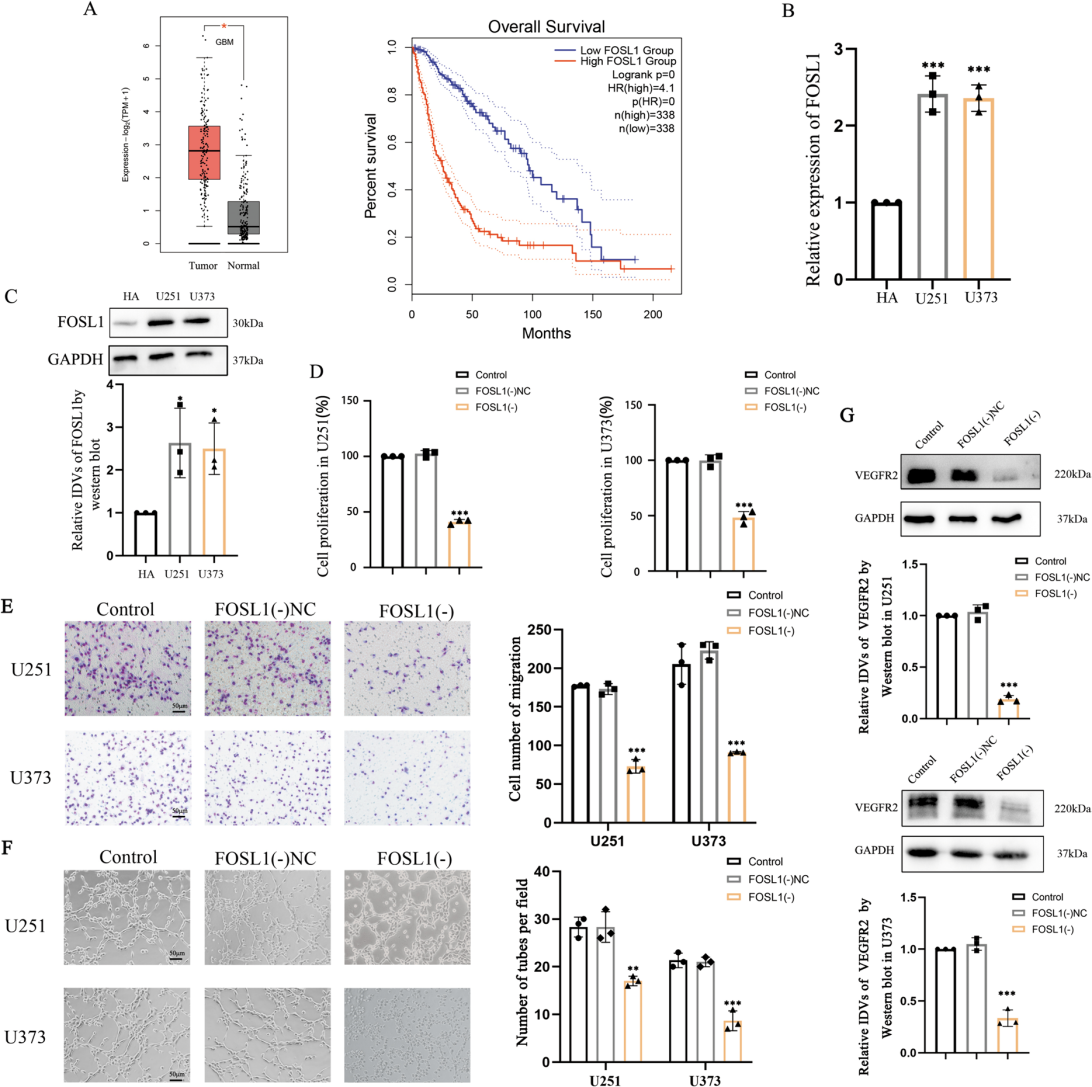
FOSL1 is significantly upregulated in GBM cells and influences VM-like behaviors. **A** Data from the GEPIA database illustrating FOSL1 expression levels in GBM and normal tissues. Compared with the normal group, ^*^p < 0.05. FOSL1 expression levels negatively correlated with survival times for glioma patients. (*p* < 0.001) **B** Relative expression of FOSL1 in HA and GBM cell lines detected by qRT-PCR. Compared with the HA group, ^***^p < 0.001. **C** Relative expression of FOSL1 in HA and GBM cells detected by Western blotting. GAPDH was used as an internal control. Compared with the HA group, ^*^p < 0.05. **D** Effects of FOSL1 knockdown on the proliferative capacity of U251 and U373 cells measured with a CCK8 assay. Y-axis plots the percentage of absorbance relative to control. Compared with the FOSL1(-)NC group, ^***^p < 0.001. **E** Transwell assays to study cell migration by U251 and U373 following FOSL1 knockdown. The chart represents the number of cells crossed. Compared with the FOSL1(-)NC group, ^***^p < 0.001. **F** Matrigel 3D cell culture assay to observe the formation of tube structures by GBM cells following FOSL1 knockdown. Compared with the FOSL1(-)NC group, ^**^p < 0.01 and ^***^p < 0.001. **G** The relative levels of immunoblotted VEGFR2 were measured by Western blot. GAPDH was used as an internal control. Compared with the FOSL1(-)NC group, ^***^p < 0.001

We next explored the mRNA expression levels for FOSL1 in the context of glioma using the CGGA database (Fig. S2B). Elevated expression of FOSL1 was predicted in GBM by the GEPIA database and high expression of FOSL1 prohibited glioma patient survival (Fig. 2A). High-expression of FOSL1 mRNA and protein in GBM was verified by qRT-PCR and Western blotting (Fig. 2B, C). To study the effects of FOSL1 knockdown on GBM cells, we constructed stably-transfected cell lines FOSL1(-)U251 and FOSL1(-)U373. Using a CCK8 cell proliferation assay, we observed that knockdown of FOSL1 resulted in the inhibition of proliferation by U251 and U373 cells (Fig. 2D). Transwell assays indicated that knockdown FOSL1 hindered cells migration (Fig. 2E), while matrigel 3D cell culture assays confirmed that knockdown FOSL1 disrupted the capacity for such cells to form tube-like structures reminiscent of VM (Fig. 2F). Using Western blotting, we found that knockdown of FOSL1 led to a significant reduction in steady-state levels of VEGFR2 (Fig. 2G).

### NFATC3 potentiates VEGFR2 expression through its transcription factor functions and by interacting with FOSL1 for DNA-binding

**Fig. 3:**
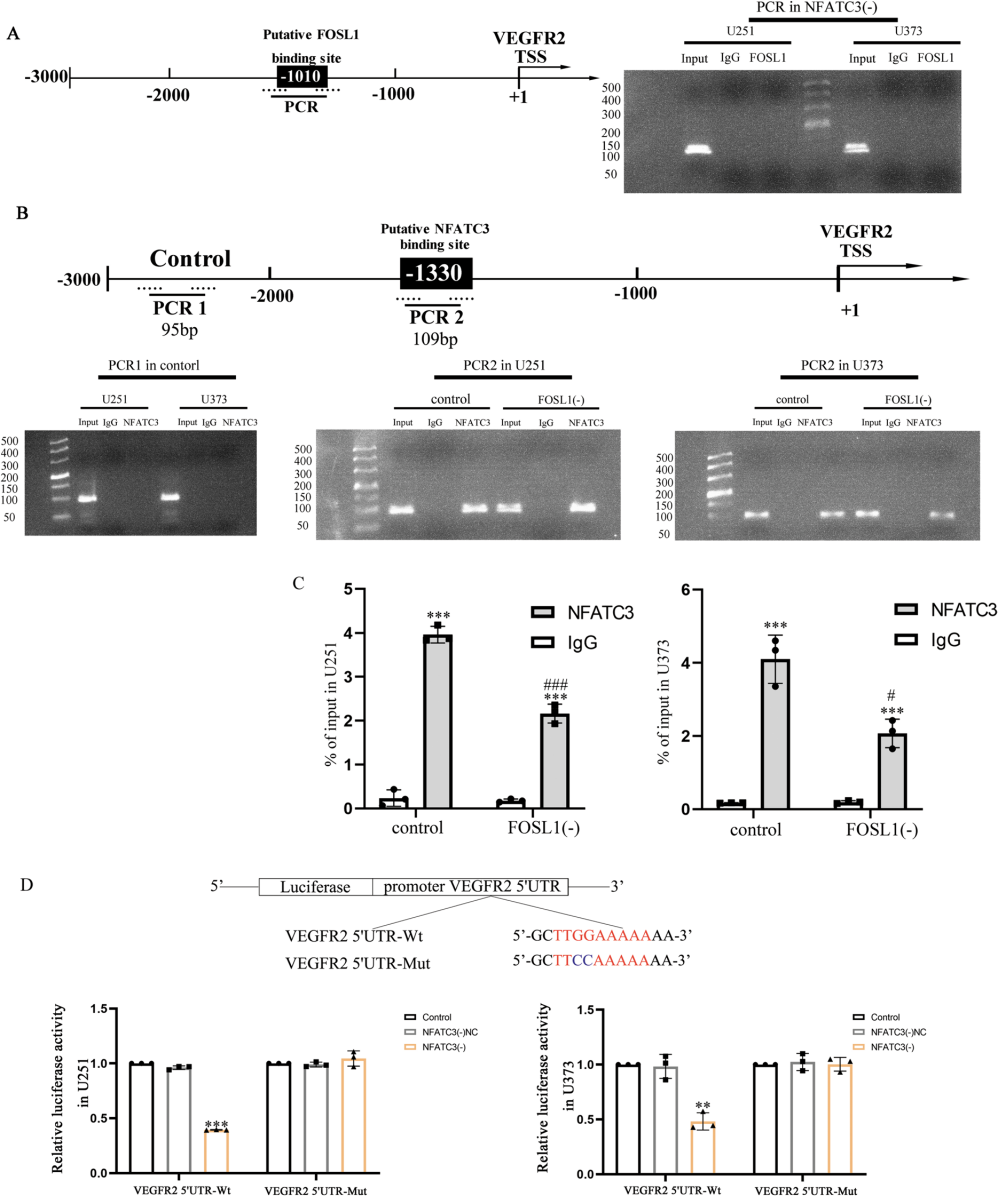
NFATC3 potentiates gene expression through binding of the VEGFR2 promoter region and interacting with FOSL1. **A** There was no ChIP evidence of binding by FOSL1 to the putative binding site of VEGFR2 in NFATC3 knockdown GBM cell lines. **B**, **C** NFATC3 was detected to bind to the VEGFR2 promoter region in normal GBM cells and FOSL1 knockdown GBM cell lines. PCR1 in Control as a negative control. Compared with the IgG group, ^***^p < 0.001; Compared with the control group, ^#^*p* < 0.05 and ^###^*p* < 0.001. **D** The effect of NFATC3 on VEGFR2 promoter activity was detected by a dual luciferase reporter assay. Compared with the NFATC3(-)NC group, ^**^p < 0.01 and ^***^p < 0.001

The VEGFR2 promoter sequence was obtained from the UCSC database, and putative binding sites of NFATC3 and FOSL1 to VEGFR2 were identified using the JASPAR database (Fig. S2C, D). We used ChIP to explore FOSL1 binding to the predicted VEGFR2 promoter site in NFATC3(-) cell lines, as well as binding of NFATC3 to predicted site within the VEGFR2 promoter in FOSL1(-) cell lines (Fig. S2E). Notably, our ChIP studies showed that FOSL1 did not bind to the predicted site within the VEGFR2 promoter in NFATC3(-) cell lines, suggesting a requirement for NFATC3 expression for binding (Fig. 3A). To further investigate the underlying molecular mechanisms, we performed ChIP experiments separately in the GBM cell lines and FOSL1(-) cell lines and found that NFATC3 binding capacity was reduced in FOSL1(-) cell lines (Fig. 3B, C). To explore this issue further, we performed dual luciferase reporter assays and found that NFATC3 bound to the promoter region of VEGFR2 and could potential transcription through this site in a DNA sequence-dependent manner (Fig. 3D). Therefore, these results suggest that NFATC3 could enhance its functions in DNA-binding and transcriptional activation by interacting with FOSL1.

### FOSL1 binds NFATC3 to co-regulate VM-like behaviors in GBM cells

**Fig. 4:**
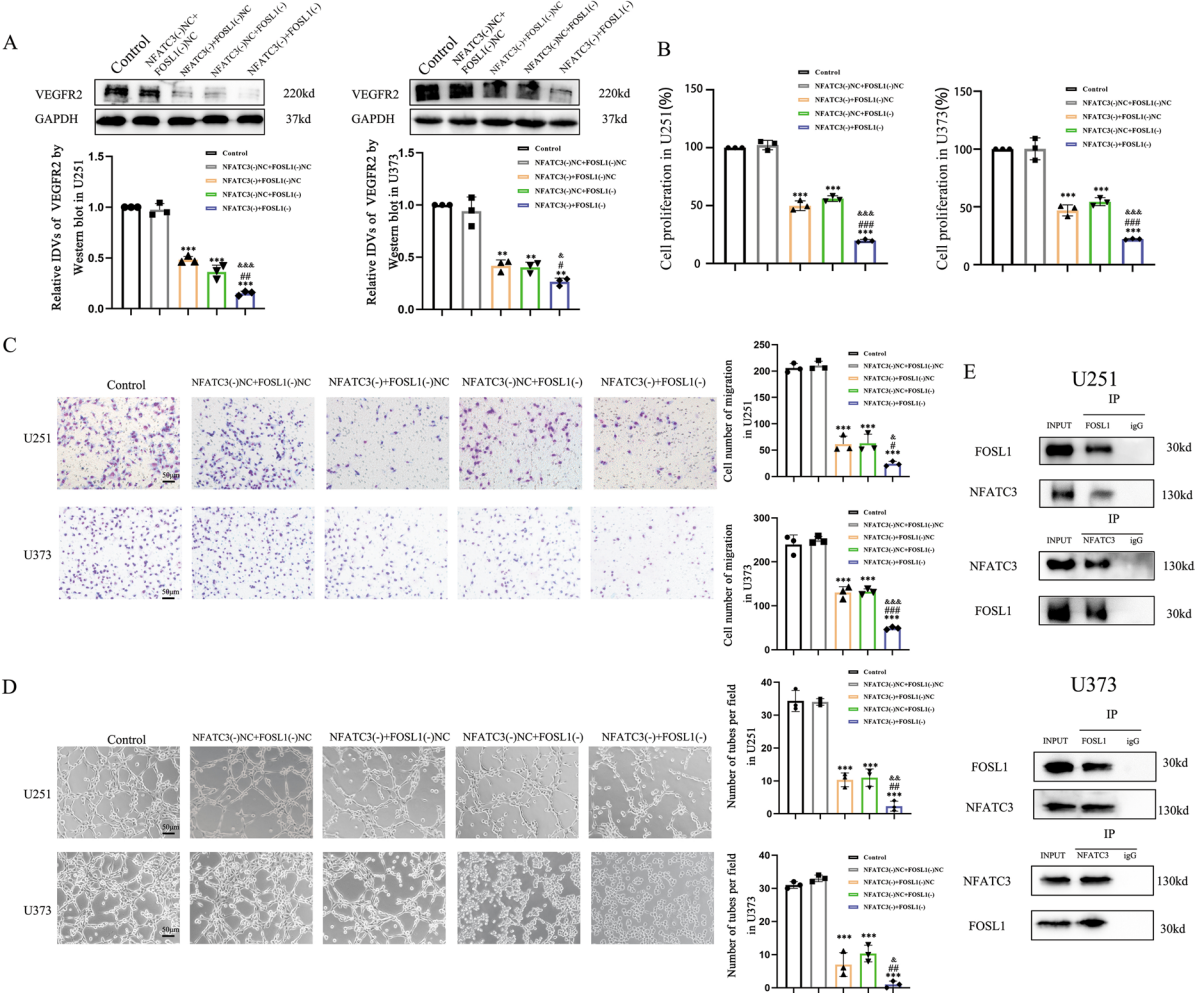
FOSL1 interacts with NFATC3 to co-regulate GBM cell traits. **A** The relative expression levels of VEGFR2 were measured across samples from each condition by Western blotting. GAPDH was used as an internal control. Compared with the NFATC3(-)NC + FOSL1(-)NC group, ^**^p < 0.01 and ^***^p < 0.001. Compared with the NFATC3(-)NC + FOSL1(-) group, ^#^*p* < 0.05 and ^##^*p* < 0.01. Compared with the NFATC3(-) + FOSL1(-)NC group, ^&^*p* < 0.05 and ^&&&^*p* < 0.001. **B-D** The effects of knockdown of both FOSL1 and NFATC3 in GBM cell lines on proliferation, migration, and tube formation. Compared with the NFATC3(-)NC + FOSL1(-)NC group, ^***^p < 0.001. Compared with the NFATC3(-)NC + FOSL1(-) group, ^#^*p* < 0.05, ^##^*p* < 0.01 and ^###^*p* < 0.001. Compared with the NFATC3(-) + FOSL1(-)NC group, ^&^*p* < 0.05, ^&&^*p* < 0.01 and ^&&&^*p* < 0.001. **E** Interactions between NFATC3 and FOSL1 were detected by co-immunoprecipitation in U251 and U373 cells

We used the bioinformatic tools GeneMANIA and STRING and found evidence of putative binding between FOSL1 and NFATC3 (Fig, S3A, B). To verify their interaction and their potential to collectively influence VEGFR2 expression, we studied U251 and U373 cells in which both FOSL1 and NFATC3 were knocked down. As shown, we found that the relative levels of VEGFR2 immunoblotted signals from Western blots using lysates from FOSL1(-) + NFATC3(-) double-knockdown cells were further attenuated in comparison with FOSL1(-) knockdown or NFATC3(-) knockdown alone (Fig. 4A). Also, we observed that knockdown of both FOSL1 and NFATC3 exacerbated the proliferation, migration, and VM formation capacities of U251 and U373, compared to knockdown of either FOSL1 or NFATC3 along (Fig. 4B-D). Given the prediction of possible binding in the string database, we performed co-immunoprecipitation experiments and found evidence of binding between NFATC3 and FOSL1 in GBM cell lines (Fig. 4E).

### HNRNPA2B1 stabilized NFATC3 mRNA by binding its mRNA sequence

**Fig. 5:**
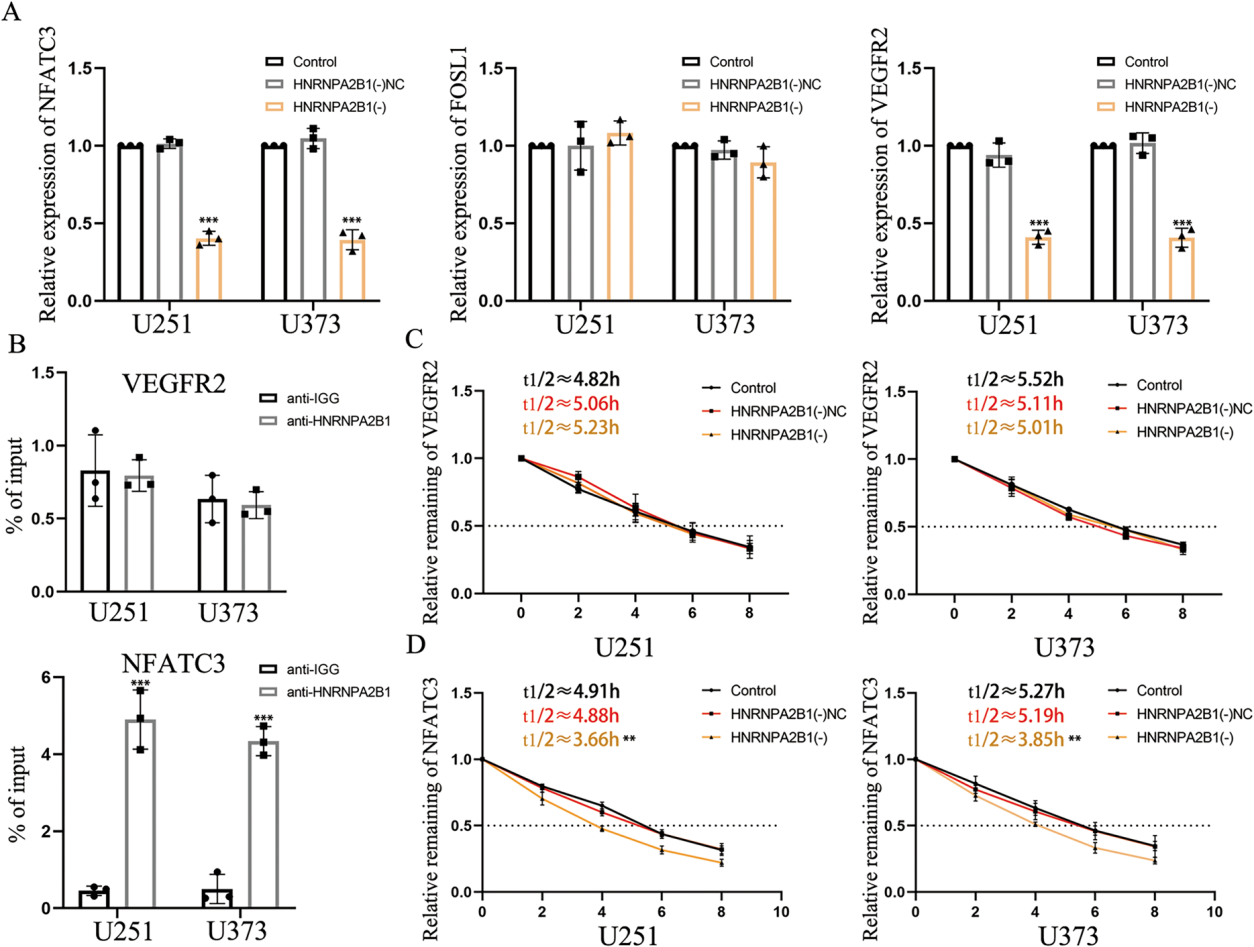
HNRNPA2B1 stabilized NFATC3 mRNA through direct binding. **A** Relative expression levels for NFATC3, FOSL1 and VEGFR2 after knockdown of HNRNPA2B1 detected by qRT-PCR assay. Compared with the HNRNPA2B1(-)NC group, ^***^p < 0.001. **B** Enrichment of VEGFR2 and NFATC3 in the anti-HNRNPA2B1 group and anti-IgG group were detected by RIP assay. Compared with the anti-IgG group, ^***^p < 0.001. **C D** After the addition of actinomycin D, the mRNA half-life of VEGFR2 and NFATC3 was detected in GBM by qRT-PCR. Compared with the HNRNPA2B1(-)NC group, ^**^p < 0.01

Evidence for HNRNPA2B1 binding to mRNAs encoding NFATC3, FOSL1 and VEGFR2 was found through a search using starBase 3.0 bioinformatics software (Fig, S3C). Moreover, mRNA expression levels for HNRNPA2B1 in different grades of gliomas were extracted from the CGGA database (Fig. S3D). We also analyzed the correlation of expression of HNRNPA2B1 with NFATC3, FOSL1 and VEGFR2 (Fig. S3E) and found that HNRNPA2B1 expression correlates with NFATC3 and VEGFR2 levels. To explore the potential inter-relationships of these factors, we performed knockdown of HNRNPA2B1 expression and quantified the relative expression levels for NFATC3, FOSL1 and VEGFR2 using qRT-PCR. As shown, NFATC3 and VEGFR2 expression levels were decreased in HNRNPA2B1 knockdown cells compared to control (Fig. 5A). To explore this further, we performed RIP assays and found that HNRNPA2B1 did not significantly influence VEGFR2 mRNAs levels in GBM cells, however it was significantly associated with NFATC3 mRNA levels (Fig. 5B). Using actinomycin D to block nascent RNA synthesis in each group of cells, we found that HNRNPA2B1 knockdown significantly shortened the half-life of NFATC3 mRNA (Fig. 5C).

### HNRNPA2B1 was significantly upregulated in GBM cells and influenced VM-like behaviors

**Fig. 6:**
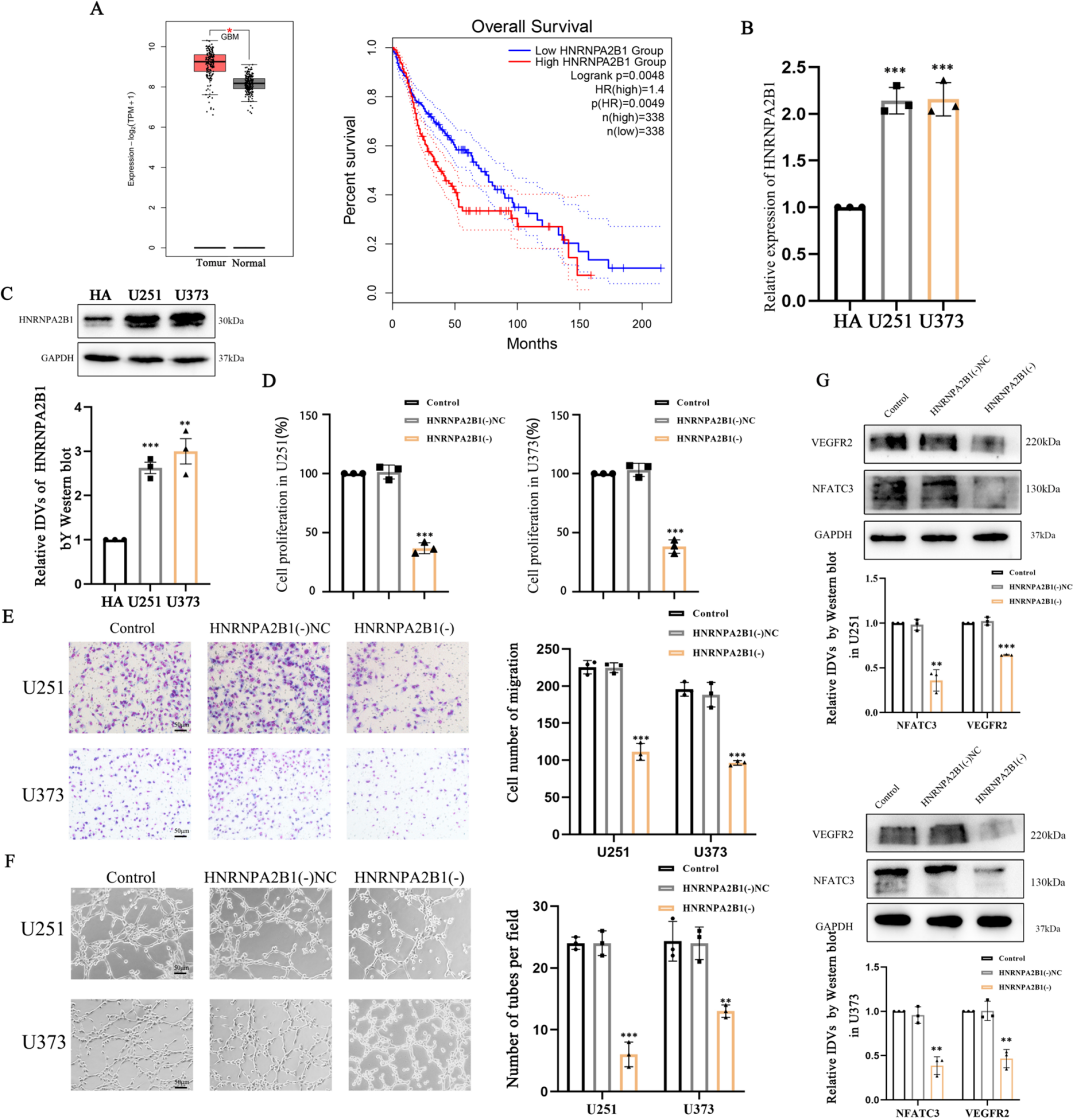
HNRNPA2B1 is upregulated in GBM, and knockdown of HNRNPA2B1 influences GBM cell behaviors. **A** Data extracted from the GEPIA database showed that HNRNPA2B1 expression levels in GBM and normal tissues. Compared with the Normal group, ^*^p < 0.05. And HNRNPA2B1 expression level on the survival time of glioma patients. (*p* < 0.01) **B** Relative expression of levels of HNRNPA2B1 in HA and GBM cells (U251 and U373) were detected by qRT-PCR. Compared with the HA group, ^***^p < 0.001. **C** Relative expression levels for HNRNPA2B1 in HA and GBM cell lines were detected by Western blotting. Compared with the HA group, ^**^p < 0.01 and ^***^p < 0.001. **D** Effect of HNRNPA2B1 knockdown on the proliferative capacity of U251 and U373 cells using a CCK8 assay. Y-axis plots the percentage of absorbance relative to control. Compared with the HNRNPA2B1(-)NC group, ^***^p < 0.001 **E** Transwell assays to study cell migration by U251 and U373 cells HNRNPA2B1 knockdown. The chart represents the number of cells crossed. Compared with the HNRNPA2B1(-)NC group, ^***^p < 0.001. **F** Matrigel 3D cell culture assay to observe tube formation ability of HNRNPA2B1 knockdown. Compared with the HNRNPA2B1(-)NC group, ^**^p < 0.01 and ^***^p < 0.001. **G** The relative expression levels of immunoblotted VEGFR2 and HNRNPA2B1 signals were measured by Western blotting. GAPDH was used as an internal control. Compared with the HNRNPA2B1(-)NC group, ^**^p < 0.01 and ^***^p < 0.001

Given the effects of HNRNPA2B1 on NFATC3 mRNA expression, we hypothesized that HNRNPA2B1 may also be relevant to the cellular behaviors of GBM cells, including their capacity to form tube-like structures in culture. As shown in Fig. 6A, we found that increasing expression of HNRNPA2B1 was correlated with reduced survival for patients with glioma, as were individuals with high expression levels for HNRNPA2B1, as documented in the GEPIA database. HNRNPA2B1 mRNA and protein were both highly expressed in GBM cells, as confirmed by qRT-PCR and Western blotting (Fig. 6B, C). By performing CCK8 assays, we found that knockdown of HNRNPA2B1 hindered the proliferation of U251 and U373 cells (Fig. 6D). Transwell assays demonstrated that knockdown of HNRNPA2B1 impaired their cell migration (Fig. 6E). Knockdown of HNRNPA2B1 also significantly disrupted the capacity for GBM cells to form tube-like structures, as shown in our matrigel 3D cell culture assays (Fig. 6F). We performed Western blot analysis to find that knockdown of HNRNPA2B1 led to a reduction in the steady levels of immunoblotted VEGFR2 and NFATC3 (Fig. 6G).

### Knockdown of NFATC3, FOSL1 and HNRNPA2B1 inhibited xenograft tumor growth and suppressed VM in nude mice

**Fig. 7:**
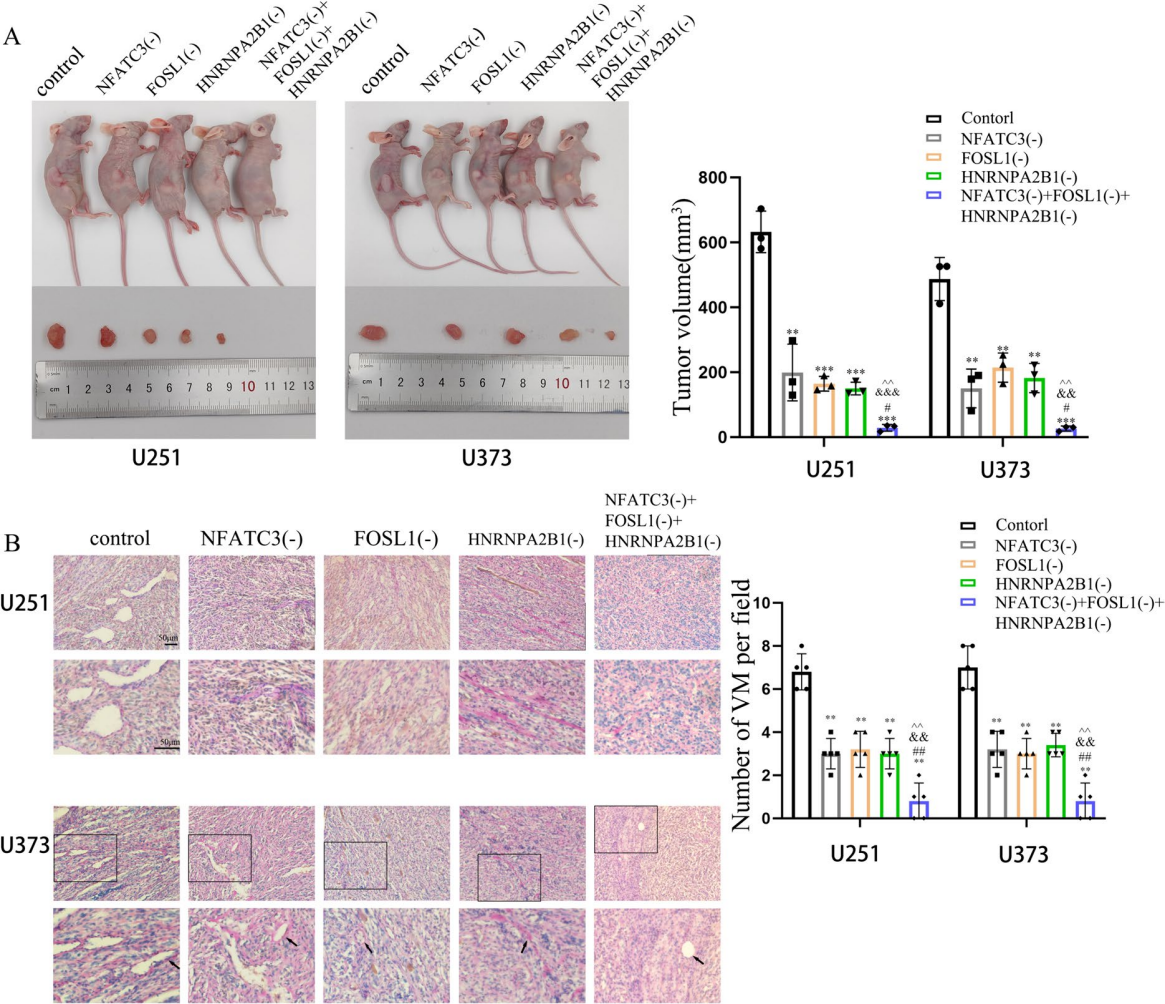
Knockdown of NFATC3, FOSL1 and HNRNPA2B1 impaired the growth of tumor xenografts and VM formation in nude mice. **A** Nude mice alongside their corresponding, excised tumor xenografts are shown. The chart represents the volumetric measurements of tumors removed for each condition. Compared with the control group, ^**^p < 0.01 and ^***^p < 0.001; compared with the NFATC3(-) group, ^#^*p* < 0.05; compared with the FOSL1(-) group, ^*&&*^*p* < 0.01 and ^*&&&*^*p* < 0.001; compared with the HNRNPA2B1(-) group, ^^^^*p* < 0.01. **B** VM in transplanted tumor sections were stained for CD31-PAS stained. Images taken at 200 × magnification. For the series of images on the upper row, a corresponding image magnified at 2X is presented correspondingly below. The black arrows point to VM structures. Compared with the control group, ^**^p < 0.01; compared with the NFATC3(-) group, ^##^*p* < 0.01; compared with the FOSL1(-) group, ^*&&*^*p* < 0.01; compared with the HNRNPA2B1(-) group, ^^^^*p* < 0.01

To verify the effects of NFATC3, FOSL1 and HNRNPA2B1 on GBM tumor growth and VM *in vivo*, we performed experiments in which U251 and U373 cells were injected under the skin of nude mice and allowed to grow for approximately 30 days. As shown, we found that the volume of GBM tumors that grew from NFATC3(-), FOSL1(-) and HNRNPA2B1(-) cells were significantly lower than control treated cells, while tumor volumes of triple-knockdown cells were further reduced (Fig. 7A). When tumors from these groups were collected, sectioned and immunostained for CD31 and using PAS to detected hallmarks of VM, we found that, compared with tumors from control cells, there was a attenuated number of VM-like channels in tumors from NFATC3(-), FOSL1(-), HNRNPA2B1(-) and NFATC3(-)/FOSL1(-)/HNRNPA2B1(-) treated cells. Moreover, sections of tumors from the NFATC3(-)/FOSL1(-)/HNRNPA2B1(-) group featured the fewest VM-like channel structures (Fig. 7B).

Based on our results, we postulate that NFATC3 in U251 and U373 cells is upregulated by HNRNPA2B1, and this leads to increased interaction between NFATC3 and FOSL1 to modulate VEGFR2 expression and positively influence VM formation in GBM.

## Discussion

Anti-vascular therapy for GBM is currently problematic because it is ineffective for GBM with VM features (Kamoun et al. 2009; Batchelor et al. 2010), and studies have shown that existing short-term effective drugs fail because of increased VM formation (Angara et al. 2017; Wu et al. 2017; Xue et al. 2017). VM-positive GBM has a lower microvascular density, and the survival time for patients is shorter than GBM without VM features (Liu et al. 2011). In this study, we have demonstrated that NFATC3 and FOSL1 are upregulated in GBM and promoted GBM migration, proliferation, and VM formation. Moreover, we describe protein–protein interaction between NFATC3 and FOSL1 and their combined actions are important for facilitating the proliferation, migration and VM-like behaviors in GBM cells, in a NFATC3-dependent manner. Also, we find that upregulation of HNRNPA2B1 enhances NFATC3 mRNA stability and can enhance NFATC3 expression. To our knowledge, our research represents the first report of the epistatic relationships and molecular interactions between HNRNPA2B1, NFATC3 and FOSL1 on VM in GBM.

Previous studies have reported that NFAT is a downstream factor for VEGFR or VEGFR2, and VEGF bound to VEGFR or VEGFR2 to activate NFAT through specific signaling pathways (Armesilla and Lorenzo 1999; Zhao et al. 2016, Ryeom et al. 2008, Alghanem et al. 2017). However, knockdown of NFATC3 influences VEGFR/VEGF expression (Graef et al. 2001). Our study shows that in GBM cells, an upregulation of NFATC3 likely leads to binding of the VEGFR2 promoter to transcriptionally activate VEGFR2 expression which, in turn, leads to an increase in VM tubes. Guided by the findings from previous studies and our current results, we speculate that there is a NFATC3-VEGFR2-NFATC3 regulatory loop that modulates VM formation in GBM. This is reminiscent of a postulated NFATC3-VEGFR2-NFATC3 signaling loop in breast cancer (Zhao et al. 2016). Furthermore, we verified that knockdown of NFATC3 inhibited the proliferation and migration capacities of GBM cells, which was consistent with previous findings (Urso et al. 2019).

FOSL1 belongs to the FOS family of transcription factors, and binds JUN to form the AP1 transcription factor complex, comprising a basic leucine zipper structure. This complex recognizes and bind DNA regulatory sequences known as TPA responsive elements (TRE) (Jiang et al. 2020), and FOSL1 can also dimerize with other transcription factors that are not AP1 members, but its function in those signaling contexts are less clear (Sobolev et al. 2022). We detected the binding of FOSL1 to NFATC3 in GBM and predicted possible binding sites for FOSL1 and NFATC3. However, in GBM cells that stably expression NFATC3 shRNA to suppress the expression of this gene, we found no evidence of binding by FOSL1 to the VEGFR2 promoter region. Furthermore, knockdown of FOSL1 in GBM cells led to reduced binding of NFATC3 to the VEGFR2 promoter. Furthermore, disruption to HNRNPA2B1 expression had no effect on FOSL1 mRNA levels. These lines of evidence indicate that FOSL1 and NFATC3 interact to transcriptionally co-regulate VEGFR2 expression in an NFATC3-dependent manner. This is consistent with the finding that NFAT interacts with the AP1 complex to promote transcription of another target, IL-13Ra2, in GBM cells (Wu et al. 2010).

HNRNPA2B1 is an RNA-binding protein that is prominent in cancer research. Elevated levels of HNRNPA2B1 are observed in gastric cancer (Hu et al. 2023), pancreatic cancer (Dai et al. 2017) and lung cancer (Yu et al. 2018), and this gene influences the proliferation and migration of cancer cells. Our knockdown studies with GMB cells also found that HNRNPA2B1 was necessary for the proliferation and migration of GBM cells. We detected that HNRNPA2B1 enriched NFATC3 mRNA rather than VEGFR2 mRNA, and knockdown of HNRNPA2B1 reduced the half-life of NFATC3 mRNA and not VEGFR2 mRNA. When we suppressed NFATC3 and VEGFR2 in stable transfected GBM cell lines using shRNAs, we found that this curtailed VM-like behaviors in GBM. These results suggest that HNRNPA2B1 influences VM-like behaviors in GBM by stabilizing NFATC3 mRNA. This is consistent with a previous study that reported that HNRNPA2B1 increases the expression of the VM marker MMP2 in GBM (Deng et al. 2016), and suggests that HNRNPA2B1 promotes VM formation in GBM through multiple pathways.

Through our in vivo experiments, we demonstrated that knockdown of NFATC3, FOSL1, and HNRNPA2B1 inhibited VM-like behaviors and resulted in a significant decrease in xenografted GBM tumors, both their sizes and volumes. Within GBM cell lines U251 and U373, we found that the regulation of VEGFR2 by HNRNPA2B1 and FOSL1 required the participation of NFATC3, such that NFATC3 was downstream of the signaling pathway. Our results are reminiscent of a study which reported that mice lacking NFATC3 showed decreased colorectal tumor numbers and reduced tumor sizes (Lin, et al. 2020). Drugs that inhibit NFAT, such as tacrolimus and cyclosporin A (CSA) that inhibit phosphatases, have demonstrated anticancer activity that is accompanied by immunosuppressive side effects (Durnian et al. 2007; Azzi et al. 2013). Considering the presence of the blood–brain barrier and phosphatase inhibition of many types of NFAT, the search for small molecule drugs that are more specific to NFATC3 could be an appropriate future direction for novel GBM treatments. This is one direction of our future research.

## Conclusion

We find that HNPNPA2B1, NFATC3 and FOSL1 are highly expressed in GBM cells and are associated with their cellular behaviors including cell proliferation, migration and the formation of tube-like structures reminiscent of VM. We find that NFATC3 promotes VM formation and transcriptionally upregulates VEGFR2 transcription. FOSL1 interacts with NFATC3 to further facilitate VEGFR2 gene expression and VM. Furthermore, the half-life of NFATC3 mRNA is prolonged by HNRNPA2B1, and NFATC3 expression is increased, ultimately leading to enhanced VM. Taken altogether, our findings suggest that targeting NFATC3 and its interacting partners could represent a viable strategy to develop novel treatments for GBM with VM features.

## Supplementary Information

Below is the link to the electronic supplementary material.

Supplementary file1 (PNG 363 KB)

Supplementary file2 (JPG 2054 KB)

Supplementary file3 (JPG 5453 KB)

Supplementary file4 (DOCX 14 KB)

## Data Availability

The datasets and materials generated and/or analyzed during the current study are available from the corresponding author on reasonable request.

## Abbreviations

VM: Vasculogenic mimicry
GBM: Glioblastoma
ChIP: Chromatin immunoprecipitation
WHO: The World Health Organization
PAS: Periodic acid–Schiff
NFAT: Nuclear factor of activated T cells
AP1: Activator protein 1
NHD: NFAT homology domain
RHD: Rel-homology domain
CDS: Calcineurin-docking site
HA: Normal human astrocytes
RIP: RNA immunoprecipitation
CO-IP: Co-Immunoprecipitation
CSA: Cyclosporin A
