# Supplementary figures and images for: HNRNPA2B1 stabilizes NFATC3 levels to potentiate its combined actions with FOSL1 to mediate vasculogenic mimicry in GBM cells
Source: Cell Biol Toxicol. 2024 Jun 11;40(1):44. doi: 10.1007/s10565-024-09890-5 (PMC11166796; doi:10.1007/s10565-024-09890-5)

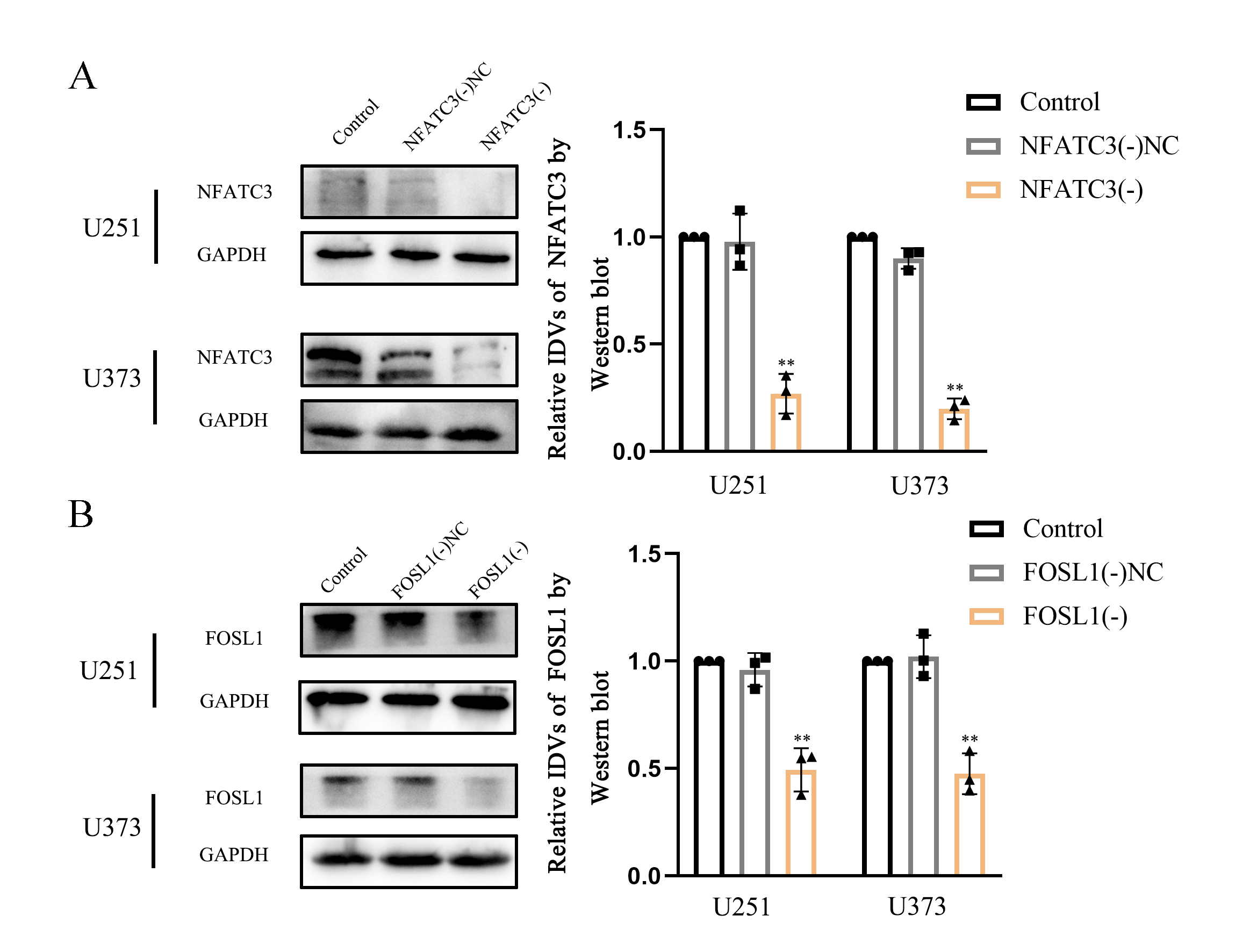

Supplement: Supplementary file 1 — Supplementary file1 (PNG 363 KB) [file 10565_2024_9890_MOESM1_ESM.png]

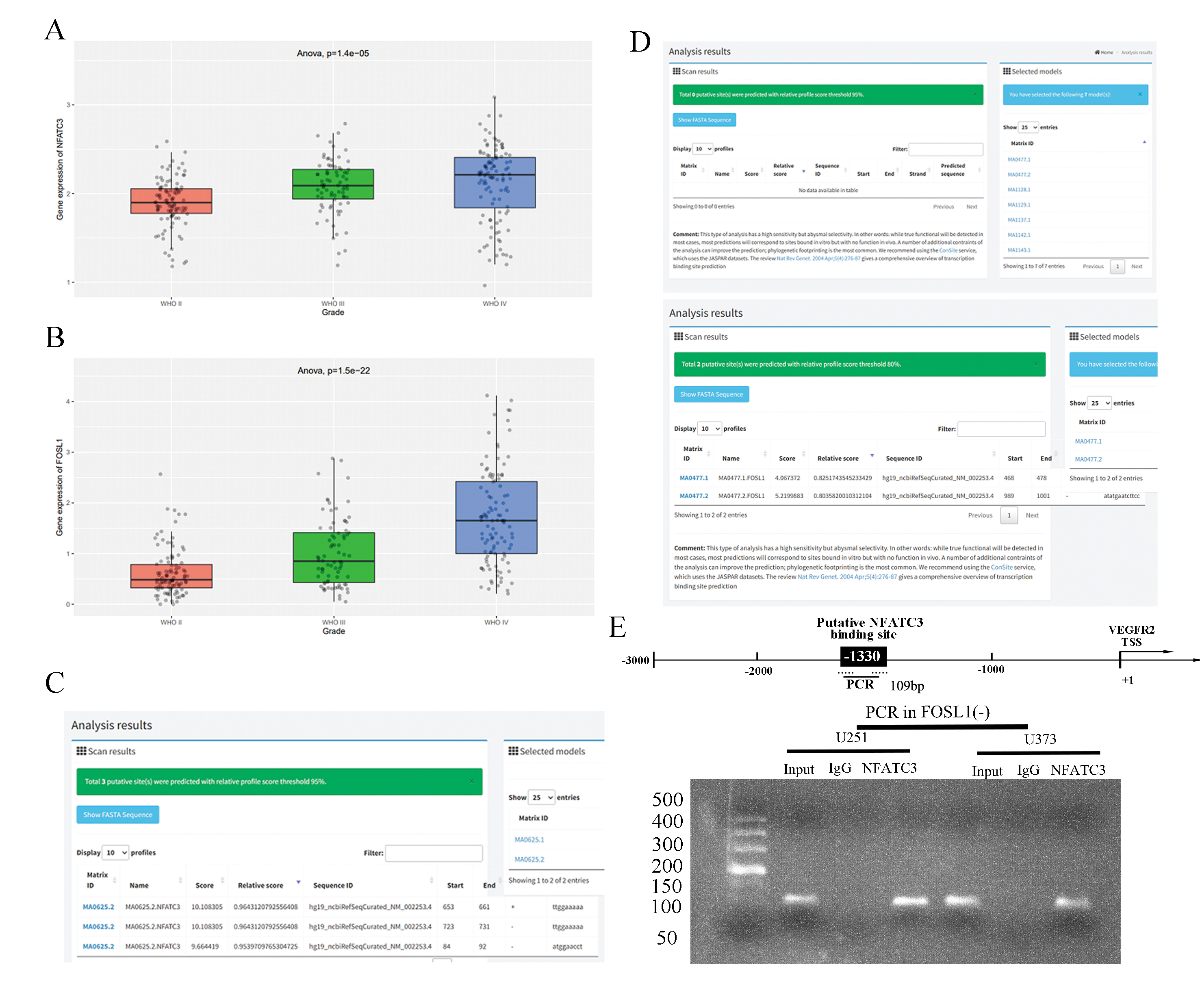

Supplement: Supplementary file 2 — Supplementary file2 (JPG 2054 KB) [file 10565_2024_9890_MOESM2_ESM.jpg]

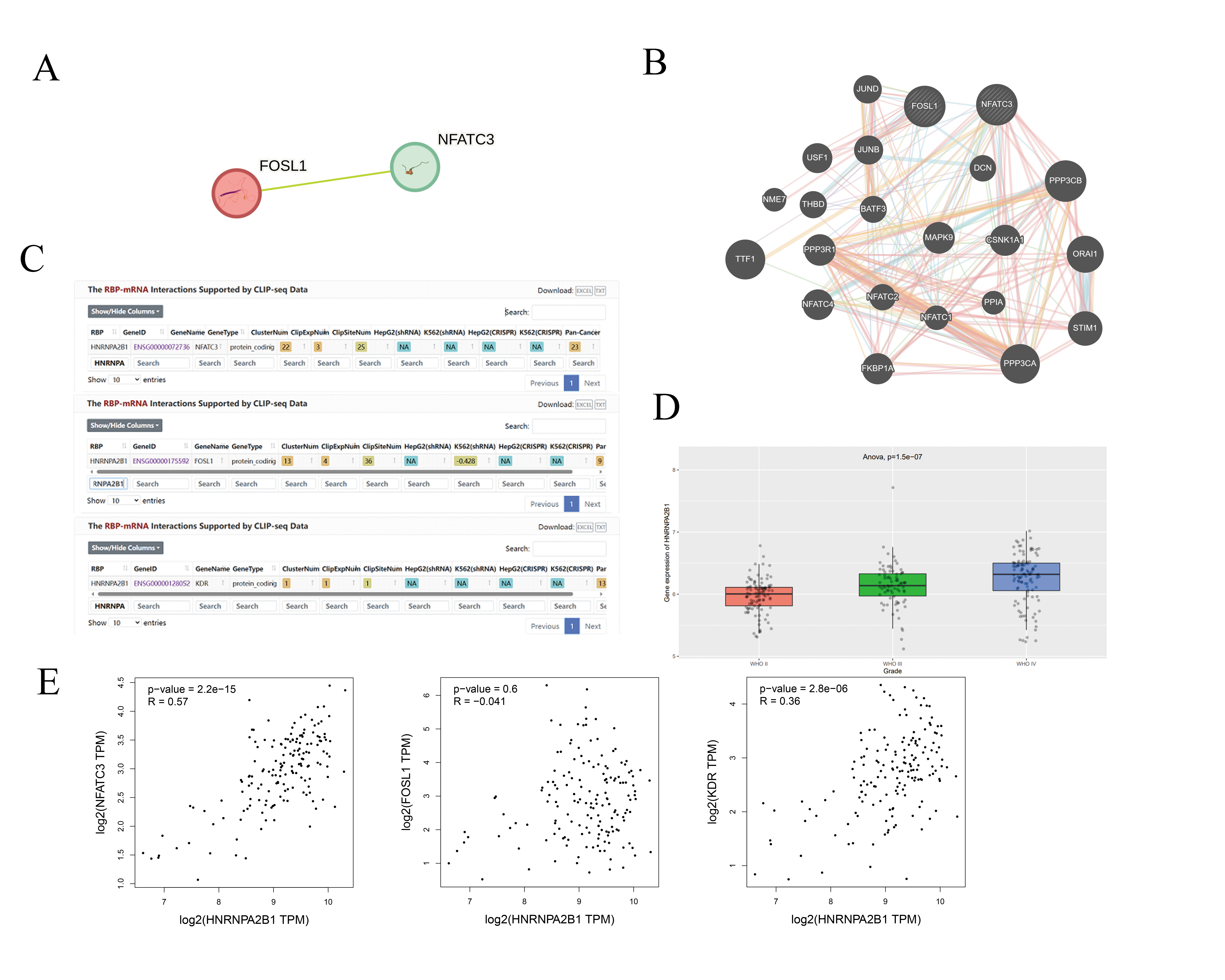

Supplement: Supplementary file 3 — Supplementary file3 (JPG 5453 KB) [file 10565_2024_9890_MOESM3_ESM.jpg]
