# Supplementary material for: HNRNPA2B1 stabilizes NFATC3 levels to potentiate its combined actions with FOSL1 to mediate vasculogenic mimicry in GBM cells
Source: Cell Biol Toxicol. 2024 Jun 11;40(1):44. doi: 10.1007/s10565-024-09890-5 (PMC11166796; doi:10.1007/s10565-024-09890-5)
Supplement: Supplementary file 4 — Supplementary file4 (DOCX 14 KB) [file 10565_2024_9890_MOESM4_ESM.docx]

**A**

Primers and probes used for qRT-PCR

| Primer or Probe | Gene | Sequence (5’->3’) or Assay ID |
| --- | --- | --- |
| Primer | VEGFR2 | F:CGGTCAACAAAGTCGGGAGA |
|  |  | R:CAGTGCACCACAAAGACACG |
|  | NFATC3 | F:GTGTACACATCCCACAGCCC |
|  |  | R:CTGAGGTCGTCCATCTTGTCC |
|  | FOSL1 | F:AGGAGCTGCAGTGGATGGTA |
|  |  | R:TACGTCTCCTGTTCACAAGGC |
|  | HNRNPA2B1 | F:TGGAGGTAGCCCCGGTTATG |
|  |  | R:GGACCGTAGTTAGAAGGTTGCT |
|  | GAPDH | F:AAATCCCATCACCATCTTCCAG |
|  |  | R:TGATGACCCTTTTGGCTCCC |

**B**

Sequences of shRNA template

| Gene | Sequence (5’->3’) | |
| --- | --- | --- |
| NFATC3 | Sense | GCTTACCACATCATGGATTAC |
| FOSL1 | Sense | CTGACAGAAGGTGCCACTTTA |

Sequences of siRNA template

| Gene | Sequence (5’->3’) | |
| --- | --- | --- |
| HNRNPA2B1 | Sense | CAGAAAUACCAUACCAUCAATT |

**C**

Primers and probes used for ChIP

| binding site detection |  | Sequence (5’->3’) or Assay ID |
| --- | --- | --- |
| NFATC3 | PCR1 | F: GATCTCAGCAGCATGAAGAAGC |
|  |  | R: ATCTTGGACATAGAAAGCACCAG |
|  | PCR2 | F: CCTCTGCCAAAAGAAAAGTCT |
|  |  | R: GACAGGTGAAATATTTTAATGCTGA |
| FOSL1 | PCR | F:GTCCATATCTCCCCACACCTG |
|  |  | R:AAGCACCTCTGGGAGGTAAAAG |

**D**

Wild-type and mutant plasmid sequences

VEGFR2

| Wild-type plasmid sequences | Mutant plasmid sequences |
| --- | --- |
| 5’GCTTGGAAAAAAA3’ | 5’GCTTCCAAAAAAA3’ |
